# Objective image analysis of real-time three-dimensional intraoperative ultrasound for intrinsic brain tumour surgery

**DOI:** 10.1186/s40349-017-0084-0

**Published:** 2017-02-16

**Authors:** Sophie J. Camp, Vasileios Apostolopoulos, Vasileios Raptopoulos, Amrish Mehta, Kevin O’Neill, Mohammed Awad, Babar Vaqas, David Peterson, Federico Roncaroli, Dipankar Nandi

**Affiliations:** 1 Department of Neurosurgery, Charing Cross Hospital, Imperial College Healthcare NHS Trust, Fulham Palace Road, London, W6 8RF UK; 2 Department of Radiology, Charing Cross Hospital, Imperial College Healthcare NHS Trust, Fulham Palace Road, London, W6 8RF UK; 3 Department of Histopathology, Charing Cross Hospital, Imperial College Healthcare NHS Trust, Fulham Palace Road, London, W6 8RF UK

**Keywords:** Brain tumours, Intraoperative ultrasound, Image analysis

## Abstract

**Background:**

There is growing evidence that maximal surgical resection of primary intrinsic brain tumours is beneficial, both by improving progression free and overall survival and also by facilitating postoperative chemotherapy and radiotherapy. Hence, there has been an increase in the popularity of real-time intraoperative imaging in brain tumour surgery. The complex theatre arrangements, prohibitive cost and prolonged theatre time of intraoperative MRI have restricted its application. By comparison, intraoperative three-dimensional ultrasound (i3DUS) is user friendly, cost-effective and portable and adds little to surgical time. However, operator-dependent image quality and image interpretation remain limiting factors to the wider application of this technique. The aim of this study was to explore objective i3DUS image analysis and its potential therapeutic role in brain tumour surgery.

**Methods:**

A prospective, observational study was undertaken (approved by the local Research and Ethics Committee prior to recruitment). Biopsies were taken from the solid, necrotic, periphery and brain/tumour interface of intrinsic primary brain tumours. Digital i3DUS images were analysed to extract quantitative parameters from these regions of interest (ROI) in the i3DUS images. These were then correlated with the histology of the relevant specimens. The histopathologist was blinded to the imaging findings.

**Results:**

Ninety-seven patients (62 males; mean 54 years) with varying gliomas (84 high grade) were included. Two hundred and ninety regions of interest were analysed. Mean pixel brightness (MPB) and standard deviation (SD) were correlated with histological features. Close correlations were noted between MPB and cellularity, and SD and intrinsic cellular diversity.

**Conclusions:**

MPB and SD are objective measures reflecting the sensitivity of i3DUS in detecting the presence and extent of intrinsic brain tumours. They indirectly suggest heterogeneity, cellularity and invasiveness, providing information of the nature of the tumour, and also reflect the sensitivity of intraoperative US to detect the presence of residual intrinsic brain tumours. Development of this paradigm will enhance i3DUS use as an adjunct in brain tumour surgery. Optimizing its intraoperative application will impact surgical resection and, hence, patient outcome.

## Background

The prognosis for patients with intrinsic brain tumours remains poor, despite advances in imaging, surgical technology and adjuvant treatments [1]. Near total resection of infiltrative neuroepithelial tumours has been shown to have a positive impact on quality of life, progression free and overall survival [2–5]. Hence, the capability to achieve maximal resection has led to an increase in the popularity of intraoperative imaging. This attempts to eliminate the error produced by brain shift, due to loss of CSF and tumour resection, an inherent problem of navigation systems based on preoperative imaging [6].

Intraoperative magnetic resonance imaging (iMRI) requires a customized operative MR suite and specialized MR compatible surgical instruments, and case duration is typically prolonged due to the time taken to acquire the images and patient transfer into and out of the scanner. However, the images produced by iMRI have excellent resolution. By comparison, intraoperative three-dimensional ultrasound (i3DUS) is a portable system offering real-time imaging, which does not require its own dedicated suite. It can be integrated into existing theatre infrastructure with minimal disruption to surgical workflow. It allows precise visualization of blood vessels, the ventricular system, lesions and immediate complications, such as a haematoma. It is also significantly cheaper. It has the potential intraoperative therapeutic use of facilitating decision-making, not purely acting as a diagnostic tool. However, ultrasound is user dependent, with an operator learning curve. Furthermore, image analysis remains an underdeveloped field.

Several neurosurgical centres have employed i3DUS, with integrated image guidance based on multimodal preoperative imaging, to achieve maximal safe resections of brain tumours [7–11]. These systems are able to update a conventional image guidance system based on preoperative images, with real-time three-dimensional ultrasound images obtained during surgery. By permitting multiple acquisitions, the frequent update of the image guidance system facilitates elimination of the inaccuracy of conventional navigation [6]. Due to variation in the information contained within each modality, the correlation of intraoperative ultrasound with preoperative MRI images has been recognized as a challenge. Various computational methods have been proposed and tested on animal and phantom models [12]. The accuracy of i3DUS navigation to target an area in the brain has been tested in vitro and recorded as within 2 mm [13]. This registration error is typical of that reported by most non-frame-based navigation systems [14] and is considered acceptable in standard clinical practice for brain tumour resection where the target is typically far larger than in functional neurosurgery. However, these studies were based on subjective interpretation of the US digital images. No study, prior to this [15], has explored the maximum potential of i3DUS as an imaging modality, using objective measures. The aims of this study, therefore, were to explore objective image analysis and its potential use in brain tumour surgery and to encourage further prospective quantitative studies to assess the accuracy and sensitivity of i3DUS to detect the true extent of intrinsic brain tumours, thus facilitating maximal resection.

## Methods

This was a prospective, observational study. The project was approved by the Local Research Ethics Committee of Imperial College Healthcare NHS Trust.

### Patients

All patients admitted to the Unit with an intrinsic brain tumour between March 2009 and November 2013 were considered for recruitment. Every patient was discussed in the neuro-oncology multi-disciplinary team (MDT) meeting, as per the standard tumour protocol. Patients undergoing biopsy or resection surgery were invited to participate. Following appropriate discussion, with provision of written information and suitable time for consideration, formal informed consent was obtained.

### Methods

The operative procedure involved the standard surgical steps for an infrared navigation system and image-guided craniotomy. The preoperative volumetric contrast-enhanced brain scan (MRI or CT), undertaken with scalp fiducials, was stored in digital format and transferred onto the SonoWand workstation (Trademark registered by the SonoWand Company Trondheim, Norway). This navigation system offers the ability to update the navigation data with sequential i3DUS sweeps taken before the dura is opened and at several stages during a biopsy/debulking procedure. Intraoperative 3DUS-guided biopsies were taken using a biopsy needle with a custom-made reference frame capable of being tracked by the SonoWand camera (a Hennig needle; Elekta). The biopsies were taken from the solid region and the periphery of the tumour and the tumour/brain interface. For high-grade lesions, they were taken from the solid and necrotic areas of each tumour and the tumour/brain interface. All biopsies were undertaken following the dural opening, prior to tumour resection. The exact site for each sample was determined at the time of the procedure by the two operating surgeons, using the preoperative scan combined with the intraoperative 3DUS image. To minimize error, the biopsies were taken prior to any resection, to ensure that the architecture of the tumour was unchanged at the time of sampling. For reference, digital images of the biopsy needle position were taken at the time of each biopsy. The specimens were subsequently examined by a histopathologist blinded to the imaging findings.

Image analysis was undertaken using ImageJ [16]. A region of interest (ROI) was defined as the area in the digital image around the opening of the biopsy needle at the time of sampling. This accommodated the distance of the aperture from the tip of the biopsy needle and was calculated to reflect the area of the image directly adjacent to the sampling site of the biopsy needle. The ROI comprised 468 pixels, which were then analysed using a brightness intensity scale (range 0–255). Mean pixel brightness (MPB) and the standard deviation (SD) of the MPB were obtained from each ROI. Further data analyses were undertaken using SPSS Statistics (Trademark registered by SPSS Statistics) and Sofastats, open-source statistical package (Trademark registered by Paton-Simpson & Associates).

## Results

Ninety-seven patients who underwent biopsy or debulking surgery were included in the analyses. There were 62 male and 35 female patients, with a mean age of 54.3 years (median 56 years). The histological diagnosis of each analysed patients is presented in Table 1.

**Table 1:** Histological diagnoses of the patient group

Tumour type	Number of patients (%)
Pilocytic astrocytoma	1 (1.0)
Diffuse astrocytoma (WHO grade II)	2 (2.1)
Oligoastrocytoma (WHO grade II)	1 (1.0)
Oligodendroglioma (WHO grade II)	9 (9.4)
Anaplastic astrocytoma (WHO grade III)	6 (6.2)
Oligoastrocytoma (WHO grade IIII)	1 (1.0)
Oligodendroglioma (WHO grade III)	6 (6.2)
Glioblastoma multiforme (WHO grade IV)	67 (69.1)
Glioblastoma with previous radiotherapy	1 (1.0)
Gliomatosis cerebri	3 (3.1)

Two hundred ninety ROIs were identified as previously detailed. There were three ROIs per patient, with the exception of the single patient with a pilocytic astrocytoma, where only two ROIs were analysed. The yield of biopsy was 100%, that is, all biopsies undertaken using i3DUS guidance produced pathological specimens. Furthermore, the samples’ descriptive histological features corresponded to the region from which they were selected (solid/necrotic/periphery/interface).

**Fig. 1:**
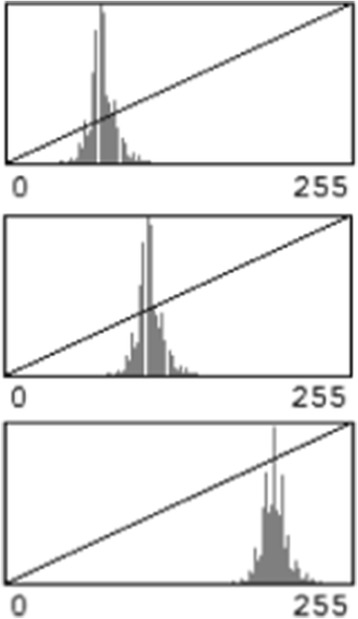
The pattern of distribution of pixel brightness at varying gain settings. The pattern of distribution of the pixel brightness was independent of the gain setting

The ultrasound gain settings used at the time of image acquisition affect the absolute values of the brightness intensity. No attempt was made to normalize the data to account for this, as obtaining maximum image quality was considered key, and the pattern of distribution of the pixel brightness was found to be independent of the gain setting, as demonstrated in Fig. 1.

Analysis of the 290 ROIs in the vicinity of the opening of the biopsy needle revealed a close correlation between the histological features of the tumours and MPB and its SD. There were also repeated recognizable patterns and characteristic of different types of tumours, as detailed below.

**Fig. 2:**
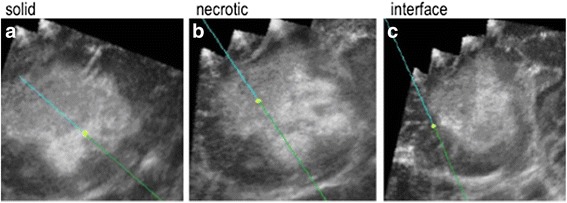
An example of the digital ultrasound images obtained from a WHO grade IV lesion. Biopsies were taken from the solid (**a**) and nectrotic (**b**) regions and the infiltrating interface (**c**) of a WHO grade IV lesion. On these ultrasound images, the *blue line* represents the biopsy needle, whilst the *yellow circle* is the tip, with a projected tip extension shown beyond the tip/yellow circle

1. Glioblastoma multiforme World Health Organization (WHO) grade IV
  In the 68 WHO grade IV patients, there were varying MPB and SD for the solid, necrotic and infiltrating interface of the tumour with the brain (Fig. 2), which were demonstrated to be statistically significantly different (Table 2). Interestingly, in 27 (39.7%) of these patients, a different pattern was noted, where the solid component showed a relatively low MPB and unusually high SD.

**Table 2:** MPB and its characteristics for the 68 patients with WHO grade IV tumours

	MPB	95% confidence intervals	SD	Minimum	Maximum
Solid	188.8	187.8–189.9	11.6	152.0	213.0
Necrotic	123.2	121.8–124.6	15.2	81.0	168.0
Interface	70.8	69.4–72.2	15.4	38.0	105.0

Paired *t* test of MPB: *p* < 0.001 (solid versus necrotic; solid versus interface)
2. Anaplastic astrocytomas WHO grade III
  In six anaplastic astrocytoma patients, biopsies were obtained from the core, that is the area of the highest echogenicity, the periphery and the tumour interface (Table 3; Fig. 3). When compared to the WHO grade IV tumours, anaplastic astrocytomas were generally found to have a low MPB at the core. At the interface with the brain, they were found to have a similar MPB.

**Table 3:** MPB and its characteristics for the six patients with WHO grade III tumours

	MPB	95% confidence intervals	SD	Minimum	Maximum
Core	166.7	165.6–167.8	12.2	126.0	208.0
Periphery	120.2	119.1–121.3	12.1	94.0	148.0
Interface	83.3	82.1–84.5	13.0	45.0	131.0

Paired *t* test of MPB: *p* < 0.001 (core versus periphery; periphery versus interface)

**Fig. 3:**
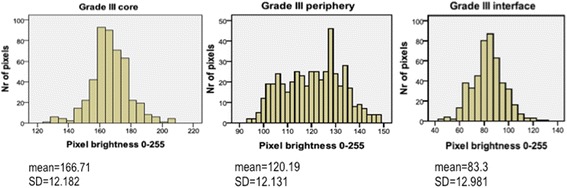
Graphic representation of MPB in the six patients with an anaplastic astrocytoma (WHO grade III). The MPB was highest at the core and lowest at the interface
3. Grade II oligodendroglioma with areas of anaplastic transformation
  In 6 of the 15 oligodendroglioma patients, anaplastic transformation was observed. In the representative case shown in Fig. 4, the preoperative scan revealed areas of enhancement. Biopsies were taken from the low echogenicity area, high echogenicity areas and the tumour/brain interface. The high echogenicity areas with the highest MPB correlated to the areas of malignant transformation (Table 4).

**Fig. 4:**
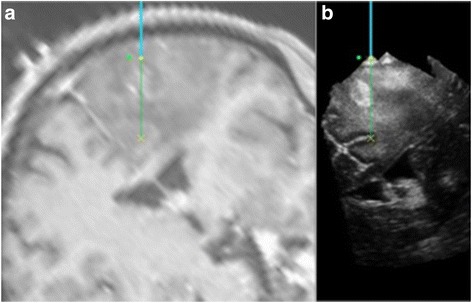
An example of an oligodendroglioma with a transformed region. The transformed region is demonstrated by the enhancement on the navigation T1 with contrast MRI (movement artefact of the patient) (**a**) and the corresponding hyperechogenic area on the i3DUS image (**b**). On these images, the navigation tool is represented by the *blue line* and its tip by the *yellow circle*. The projected tip extension is shown as a continuation of this line

**Table 4:** MPB and its characteristics for the six patients with oligodendroglioma and areas of anaplastic transformation

	MPB	95% confidence intervals	SD	Minimum	Maximum
WHO grade II region	122.2	121.0–123.4	13.5	88.0	162.0
Transformed region	182.2	182.2–183.7	16.9	123.0	228.0
Interface	31.5	30.2–32.7	14.1	10.0	110.0

Paired *t* test of MPB: *p* < 0.001 (grade II versus transformed; grade II versus interface)
4. Infiltrative astrocytomas WHO grade II
  Low-grade infiltrative astrocytomas were found to have consistently lower MPB in the centre and periphery (85.8 and 77.8, respectively). The MPB at the interface of the low-grade infiltrative astrocytomas was 61.5.
5. Pilocytic astrocytoma WHO grade I
  The analysis of one pilocytic astrocytoma showed the interface SD lower than the core SD of the tumour. Table 5 details the MPB and SD.

**Table 5:** MPB and its characteristics for the patient with a pilocytic astrocytoma

	MPB	95% confidence intervals	SD	Minimum	Maximum
Core	124.5	123.2–125.8	14.1	94.0	168.0
Interface	38.1	37.1–39.2	11.5	20.0	78.0

Paired *t* test of MPB: *p* < 0.001 (core versus interface)

To explore the specificity of i3DUS, the imperial tumour index (ITI) was computed (MPB/SD of that MPB). The ITI was proportional to cellularity and was raised in solid areas of high-grade tumours, as detailed in Table 6.

**Table 6:** The imperial tumour index for the different tissue types

	MPB	SD	ITI
Grade IV solid	188.8	11.6	16.3
Grade IV necrotic	123.2	15.2	8.1
Grade IV interface	70.8	15.4	4.6
Grade III core	166.7	12.2	13.7
Grade III periphery	120.2	12.1	9.9
Grade III interface	83.3	13.0	6.4
Grade II astrocytoma core	85.8	10.3	8.3
Grade II astrocytoma periphery	77.8	11.4	6.8
Grade II astrocytoma interface	61.5	10.4	5.9
Oligodendroglioma transformed	182.2	16.9	10.8
Oligodendroglioma grade II	122.2	13.5	9.1
Oligodendroglioma interface	31.5	14.1	2.2
Pilocytic astrocytoma core	124.5	14.1	8.8
Pilocytic astrocytoma interface	38.1	11.5	3.3

The infiltration index was also calculated as the ratio of the SD of the core or solid component MPB: the SD of the interface MPB. The infiltration index (II) was found to be less than 1 in infiltrative tumours and higher than 1 in noninfiltrative tumours, that is, the pilocytic astrocytoma (Table 7).

**Table 7:** The infiltration index for the different tissue types

	SD core or solid component	SD interface	Infiltration index
Grade IV	11.6	15.4	0.75
Grade III	12.2	13.0	0.94
Grade II astrocytoma	10.3	10.4	0.99
Oligodendroglioma grade II	13.5	14.1	0.96
Pilocytic astrocytoma	14.1	11.5	1.23

## Discussion

This prospective study has provided preliminary data of i3DUS image analysis. Several studies have already demonstrated the benefits of intraoperative real-time US for achieving maximal safe resection in brain tumour surgery [7–11]. This study succeeds previous work, showing the use of objective image analysis in determining the presence and the extent of intrinsic brain tumours, which has the potential to guide resection. A close correlation between the histological features of intrinsic primary brain tumours and MPB and its SD is reported, with recognizable patterns and characteristic of different types of tumours.

MPB objectively quantifies echogenicity and SD heterogeneity. The solid part of high-grade gliomas was found to have the highest MPB, whilst lower MPB readings were observed at the necrotic core of the tumours. Low MPB values were also consistently observed at the infiltrating margins of the tumours. SD was higher at the infiltrating margins of the high-grade tumours. It was also elevated within the same tumours in areas of varying cellularity and within solid high-grade gliomas (WHO grade IV) with impending necrosis. A possible explanation for these observations is that these different cell types produce a heterogenous population. This intrinsic diversity is reflected in the tissues’ physical properties, with varying echogenic characteristics, which are visualized by US. In comparison, hypercellular regions, such as the solid part of high-grade glioma WHO grade IV, comprise densely packed homogenous malignant cells (hence, the high MPB) and relatively low SD.

This preliminary work has shown MPB as a surrogate measure of tissue density, which in turn is a sensitive indicator of cellularity, and SD as a measure of cellular diversity. US is a mechanical wave which is altered as it travels through tissue (attenuation, absorption, refraction, reflection). It is very sensitive at detecting pathology which alters the physical properties of the brain parenchyma, even if these changes are very modest. The use of image analysis in this study eliminated the subjective interpretation of US and demonstrated the sensitivity of i3DUS in identifying regions of higher cellularity within intrinsic brain tumours and their extent. This may be of particular use in low-grade gliomas. The US data may also be able to demonstrate necrosis before it is apparent on CT/MRI brain scans. Furthermore, SD at the margins of a lesion, when sampled and analysed in real time, may provide the surgeon with information regarding a possible surgical plane. These findings illustrate the application of i3DUS and its potential therapeutic use during surgical resection.

The ITI attempted to quantify the differences in the distribution of MPB, which were then assessed in relation to tissue type. ITI was highest in solid regions of high-grade lesions but was not found to be specific for different tumour types. The infiltration index was inversely related to the infiltrative potential of the tumour type. The lowest infiltration indices were observed in grade IV lesions, with that greater than 1 in the lesion with no infiltrative potential. This is again a preliminary work on a relatively small data set and requires further development. The role of 5-aminolevulinic acid in high-grade tumours and intraoperative MRI in low-grade lesions are acknowledged, and with augmentation, i3DUS is not envisaged as an alternative. However, it is hoped that further development of this field will expand the armamentarium of the neurosurgeon.

In this study, patients with suboptimal imaging were excluded, which created a bias. However, the aim of this study was to provide preliminary image analysis data, and therefore, only the highest quality images were selected. The careful choice of targets and the exclusion of data, where there was any uncertainty of the clarity of the imaging, aided the avoidance of artefacts.

Image analysis was undertaken using ImageJ, a Java-based open source software, which has been validated in image analysis for electron microscopy in microbiology, genetics and more recently in the analysis of images taken by powerful telescopes in astronomy [16]. MPB is an established measure to quantify echogenicity, which in turn is representative of the characterization of the parenchyma [17, 18]. Indeed, relative pixel brightness has been used as an objective parameter for the quantification of the echogenicity of ROIs in neonatal brains [19].

The results from the current study are significant in the context of the growing evidence that the extent of resection of intrinsic brain tumours impacts clinical outcome [2–5]. Hence, there is a role for highly sensitive, accurate and affordable intraoperative guidance. Artefacts are an acknowledged limitation of US imaging [20]. They are more prominent in tumour resection due to the mirroring/shadowing effect of the tumour cavity. This may be overcome by modern, smaller probes which can be introduced into the surgical cavity, permitting scanning of the walls. The direct contact may minimize such artefacts. Furthermore, a profile of the characteristics of such artefacts will be gathered over time.

## Conclusions

Historically, the use of i3DUS for brain tumour surgery has suffered from a lack of objective image interpretation. This study aimed to address the matter by providing preliminary data. This work demonstrated the ability of i3DUS to detect the presence and distinct properties of intraparenchymal brain tumours, with close correlations between imaging characteristics and histological features. Based on the findings of this prospective study, there is potential to further advance i3DUS with sophisticated image analysis techniques, in order to achieve affordable, reliable, portable, user-friendly, real-time, intraoperative image guidance and analyses which will promote the therapeutic option of surgery in the treatment of brain tumours.

## Abbreviations

i3DUS: Intraoperative three-dimensional ultrasound
II: Infiltration index
iMRI: Intraoperative magnetic resonance imaging
ITI: Imperial tumour index
MDT: Multi-disciplinary team
MPB: Mean pixel brightness
ROIs: Regions of interest
SD: Standard deviation
WHO: World Health Organization
